# Facilitated Intubation: Time to Re-examine an Old Technique With Its Associated Risks Mitigated by New Technology

**DOI:** 10.7759/cureus.43364

**Published:** 2023-08-12

**Authors:** Joshua B Lowe, Michael J Yoo, John O Patrick, Rachel E Bridwell

**Affiliations:** 1 Emergency Medicine, Brooke Army Medical Center, Fort Sam Houston, USA; 2 Emergency Medicine, Royal Air Force (RAF) Lakenhealth Medical Center, RAF Lakenheath, GBR; 3 Emergency Medicine, Madigan Army Medical Center, Joint Base Lewis-McChord, USA

**Keywords:** video laryngoscopy, bilevel positive pressure ventilation, first-pass success, semi fowler positioning, airway management, critical care, covid-19, difficult airway, facilitated intubation

## Abstract

Background: Facilitated intubation (FI) refers to intubation performed using a sedative or anesthetic drug as an induction agent, without the use of a paralytic (neuromuscular blocking agent). In comparison, rapid sequence intubation (RSI) employs both an induction agent and a paralytic drug. RSI has been seen to outperform FI in terms of first-pass success when performing direct laryngoscopy and was quickly adopted as the gold standard in all situations. Recently, ketamine-only intubation has been used in situations where there is distorted anatomy or apnea intolerance (physically and physiologically difficult airways) resulting in an increased risk of a can’t intubate/can’t oxygenate scenario or significant hypoxemia. Frequent and recurring national ketamine shortages have resulted in renewed interest in whether or not other forms of FI are feasible in an era where other factors that mitigate complictions in achieving first-pass success (video laryngoscopy, bougie use, semi-Fowler positioning) are commonly used. We present a case series with outcomes for profoundly hypoxic patients with coronavirus disease 2019 (COVID-19) (physiologically difficult airways) undergoing FI during a time of national ketamine shortage, using modern techniques and technology to maximize first-pass success and minimize peri-intubation complication.

Methods: We included patients with COVID-19 pneumonia with pre-intubation oxygen saturations of less than 80% (significant hypoxemia) requiring intubation who presented to a tertiary care center in southern United States between August 25, 2021, and October 22, 2021. In this specific cohort, patients underwent endotracheal intubation with midazolam for induction without the use of paralytic agents. We used video-assisted laryngoscopy to increase the success of the first-pass attempt as well as placing the patients in a semi-Fowler position (head of bed elevation 30-45°) and bilevel positive pressure pre-oxygenation to minimize peri-intubation complications.

Results: Our case series included 29 consecutive patients that met the inclusion criteria. The mean ± standard deviation (SD) age of the patients was 49.5±15.0 years. The mean±SD pre-intubation oxygenation of our cohort was 73.1±5.9%. All 29 intubations were successful on the first-pass attempt. Only one patient (3.4%) required a rescue paralytic to facilitate oral opening. Of note, 27/29 (93%) of the patients did not receive any immunizations (including partial) for COVID-19. There were no incidents of peri-intubation arrest (cardiac arrest within 30 minutes of induction) or aspiration.

Conclusions: In 29 physiologically difficult patients with acute respiratory failure, in whom the physician determined that RSI posed a higher than normal risk, FI assisted by VL, semi-Fowler positioning, and bilevel positive pressure pre-oxygenation resulted in excellent successful first-pass intubation rates without any incidences of peri-intubation arrest or aspiration. While this cohort was small, our study reveals that FI with midazolam does not likely pose a higher risk than ketamine-only intubation and warrants further study.

## Introduction

Since the late 1990s, rapid sequence intubation (RSI) has been the technique of choice for urgent/emergent endotracheal intubation (ETI). RSI is the near-simultaneous administration of a sedative agent and neuromuscular blocking agent (NMBA). The Cochrane systematic review reports that neuromuscular blockade and RSI have been shown to have increased first-pass success and decreased peri-intubation complications associated with emergent/urgent ETI compared with intubation facilitated by a sedative alone. The primary complications reported were decreased first-pass success rate and patient reported discomfort; primarily defined as sore throat and hoarseness. However, the authors did classify the quality of evidence as low due to “indirectness, heterogeneity, and high or uncertain risk of bias” [1].

In situations where there is distorted anatomy or apnea intolerance resulting in an increased risk of a can’t intubate/can’t oxygenate scenario or significant hypoxemia, the intubating physician may deem the patient not not suitable for neuromuscular blockade. Most commonly, this patient population has undergone an “awake intubation”, with minimal to no sedation [2,3]. Recently, there has been an increase in "ketamine-only" intubations in this patient population to provide dissociative sedation while preserving airway protective reflexes and spontaneous ventilation without the adverse effects of NMBAs [2,4]. The rate of first-pass success in ketamine-only intubations has been equivalent to those noted in FI (63-73%) and significantly less than RSI (73-90%) [2,4], though the rate of complications for both ketamine and FI have not been significantly different than with RSI (16% vs 13% vs 12%, respectively) [2,4].

Coronavirus disease 2019 (COVID-19) is a contagious illness caused by the severe acute respiratory syndrome coronavirus 2 (SARS-CoV-2). This virus leads to a wide manifestation of symptoms and complications from mild upper respiratory symptoms to more serious complications such as severe acute respiratory distress syndrome (ARDS). Compared to rapid sequence endotracheal intubation (ETI) in patients without COVID-19, ETI in patients with COVID-19 carries a higher risk of adverse events (up to 40%) and peri-intubation cardiac arrest rate (as high as 4%) [5,6]. This suggests that the risk of peri-intubation cardiac arrest is nearly four times as high as patients undergoing RSI without COVID-19 [1]. Furthermore, this suggests the physiologic fragility of patients with COVID-19 needing mechanical ventilation, especially during the induction and peri-intubation period [5,6]. Although the exact mechanism of the increased risks of peri-intubation adverse events is unclear, critically ill patients with COVID-19 are likely hemodynamically labile, acidemic, and have a poor respiratory reserve [5,6]. Additionally, these risk factors are exacerbated by a depleted catecholamine response and refractory hypoxemia, providing little to no hemodynamic compensatory abilities [5,6]. 

These physiologic challenges have led to the use of non-invasive oxygenation, such as high-flow nasal cannula and non-invasive positive pressure ventilation (NIPPV), and awake proning to delay or even avoid ETI in patients with severe COVID-19 [1]. Furthermore, increased mortality associated with ETI that was realized early in the pandemic shifted respiratory strategies to rely more on non-invasive modalities which resulted in decreasing the overall incidence of ETI [3]. Importantly, this approach does not appear to have a detrimental effect on mortality [4]. Due to the success of non-invasive therapies, the patients with COVID-19 still requiring ETI represent a unique cohort who have failed conservative management with extraordinarily tenuous physiology [4]. This patient population represents one that the intubating provider may determine to be unsuited for the use of an NMBA as they have been associated with hypotension and dysautonomia, and therefore may elect to pursue ketamine-only intubations.

In the summer and fall of 2021, the southern United States experienced a surge of COVID-19 cases attributed to the Delta variant (B.1.617.2), which had a particularly strong predilection for the lower respiratory tract that resulted in the profound hypoxemia mentioned above. At the same time, there was a national shortage of ketamine. We present a case series of profoundly hypoxic patients who underwent urgent ETI during this time period and were intubated using facilitated intubation using midazolam and video-assisted laryngoscopy (VL) to mitigate the decreased first-pass success typically associated with FI.

## Materials and methods

Study design and setting

This study was reviewed and approved by the Component Office for Human Research Protections (COHRP), the Defense Health Agency (DHA) Office of Research Protections (ORP) (DHQ#: DHQ-22-2008; EIRB#: 945505). We conducted a retrospective case series of 29 profoundly hypoxic patients requiring intubation with COVID-19. The patients included in this study were admitted to a tertiary care center in the southern United States between August 25, 2021, and October 22, 2021. The intubations were performed by residency-trained emergency medicine physicians who provided nighttime coverage for rapid response and codes for admitted COVID-19 patients. The location of the intubations varied between the emergency department (ED), a step-down unit, and an intensive care unit (ICU). 

Study population

We included adult patients, aged 18 years of age or older, with laboratory-confirmed COVID-19, defined as a positive result on a reverse transcriptase-polymerase chain reaction (RT-PCR) assay. Additionally, all patients were evaluated by an emergency physician, deemed to have failed conservative management, and required mechanical ventilation. Patients included in this study were severely hypoxic with pre-intubation oxygen saturations (SpO2) ≤ 80%, who the physician determined were at high risk of peri-intubation complications and would benefit from intubation without the use of NMBAs. Exclusion criteria included prisoners and children (younger than 18 years of age).

Intubation protocol

All intubations in this study were performed by residency-trained emergency physicians. Patient positioning was semi-Fowler (non-supine, with head of bed elevation of 30-45°). All patients underwent pre-oxygenation with bilevel NIPPV for a minimum of 10 minutes at 100% fraction of inspired oxygen (FiO2). VL was used to optimize first-pass success. The VL device (C-MAC® (Karl Storz SE & Co. KG, Tuttlingen, Germany) or GlideScope (Verathon Inc., Bothell, Washington, United States)), blade type (hyper-angulated vs "regular"), and size were at the intubating provider's personal preference. Due to a national and hospital shortage of ketamine, the induction agent used was midazolam (0.02-0.04 mg/kg). The treatment team maintained a “rescue” paralytic (1 mg/kg of rocuronium) in the event of a masseter spasm or laryngospasm.

Data collection

De-identified intubation data were collected by performing a chart review from the institution's electronic medical record system at the time of intubation. We collected data regarding intubation attempts, aspiration events, and the requirement of rescue paralytic. Peri-intubation cardiac arrest is defined in this study as cardiac arrest within 30 minutes of induction that resulted in the activation of resuscitation in the form of cardiopulmonary resuscitation (CPR) or, if the patient had a do not resuscitate (DNR) order, then the loss of pulses. Furthermore, we obtained demographic data, vaccination status, comorbidities, and vital signs.

Statistical analysis

We summarized all data using descriptive statistics. Descriptive statistics included means and SD for continuous variables and counts with percentages for categorical variables. We did not impute any missing data. We used Excel (Microsoft Corporation, Redmond, Washington, United States) for all database management.

## Results

We identified 29 profoundly hypoxic patients with confirmed COVID-19 who were intubated between August 25, 2021, and October 22, 2021, using facilitated intubation. The patient characteristics are given in Table 1.

**Table 1:** Patient Characteristics SpO2: Saturation of Peripheral Oxygen

Characteristics	Values
Age (years), mean± SD (range)	49.5±15.0 (25-74)
SpO2 (%), mean ± SD (range)	73.1±5.9 (56-80)
Sex, n (%)	
Male	19 (66)
Female	10 (34)
Comorbidities, n (%)	
Obesity	9 (31)
Morbid obesity	5 (17)
End-stage renal disease	2 (7)
Diabetes mellitus	2 (7)
Lupus nephritis	1 (3)
Human immunodeficiency virus	1 (3)
Vaccination status, n (%)	
Full	2 (7)
Partial	0
Unvaccinated	27 (93)
Successful Attempt, n (%)	
1st pass	29 (100)
2nd pass	0
>2 attempts	0
Unsuccessful	0
Peri-intubation arrests, n (%)	0
Aspiration events, n (%)	0
Rescue paralytic, n (%)	1 (3)

The mean±SD age of the patients was 49.5±15.0 years with a range of 25-74 years of age, and 19/29 (65.5%) of the patients were male. The mean±SD maximum SpO2 of the patients in our study was 73.1±5.9% with a range of 56-80%. Most of the patients (27/29, 93%) were unvaccinated. The two vaccinated patients in this study had significant comorbid conditions; one had end-stage renal disease secondary to lupus nephritis requiring intermittent hemodialysis, and the other was positive for HIV. Obesity (body mass index (BMI) between 30-40 kg/m^2^)(31%) and morbid obesity (BMI >40 kg/m2)(17%) were the most common comorbidities.

No patients (0/29) experienced peri-intubation cardiac arrest or aspiration events during the FI attempt while using the study’s protocol. All FIs (29/29) were successful on the first-pass attempt. One patient (3.4%) developed a masseter spasm prior to the first attempt, requiring the administration of a rescue paralytic. 

## Discussion

We developed a FI airway management protocol to mitigate the physiologically challenging aspects of intubating critically ill patients. Ketamine-only intubation has become a common approach used to manage physically and physiologically difficult airways as the use of NMBAs may put these patients at serious risk of peri-intubation hypoxemia due to rapid de-recruitment and minimal to no pulmonary reserve [5,6]. Unfortunately, during the study period, there was a national ketamine shortage. Subsequently, we attempted to prevent complications by performing FIs. We attempted to optimize first-pass success by the use of VL, patient positioning, and maximizing bilevel positive pressure pre-oxygenation. We experienced generally excellent peri-intubation outcomes in an incredibly ill cohort of patients with COVID-19 with the risks associated with the use of FI properly mitigated with the use of modern technologies and techniques.

FI

The use of neuromuscular blocking agents, both depolarizing and non-depolarizing, has repeatedly proven to facilitate first-pass success in emergent ETI [1,2]. Because profound hypoxia and minimal physiologic reserve are associated with COVID-19, optimization of expedited first-pass intubation is critical. RSI, the administration of both sedative and paralytic in rapid succession, is considered the standard of care, for a good reason. It has been shown to increase intubation success, decrease the number of attempts required to intubate, and decrease aspiration events, though there has been no difference in mortality [7,8]. However, the use of neuromuscular blocking agents may be associated with serious adverse effects [9]. Specifically, due to their effects on muscarinic receptors and histaminergic release, these drugs produce undesirable cardiovascular effects including both bradycardia, tachydysrhythmias, and hypotension [10]. Additionally, due to their blockade of nicotinic receptors on the autonomic ganglia and adrenal medulla, these medications can produce dysautonomia [10]. In a small subset of patients who pose a physically or physiologically difficult airway in which the use of an NMBA may pose an increased risk, FI (which until recently has only been performed with ketamine) is an appropriate strategy, the downsides of which appear to be mitigated with the use of the following techniques.

VL

Several studies have demonstrated an increased rate of first-pass success in ETI when using VL compared to direct laryngoscopy, possibly due to improved laryngeal views regardless of features classically associated with difficult airways and decreased need for external laryngeal manipulation [11-14]. Secondarily, VL offers infection control specific benefits, to include an increased distance between the airway of the patient and the intubating provider and no degradation of visibility when wearing personal protective equipment [14]. In our case series, first-pass success was optimized by the use of VL while employing an FI approach to mitigate the adverse effects of NMBAs.

Semi Fowler Positioning

Semi-Fowler/head-up positioning is a technique that increases both pre-oxygenation and first-pass success in morbidly obese patients requiring endotracheal intubation [15,16]. A recent National Emergency Airway Registry (NEAR) study demonstrated similar first-pass success comparing supine versus non-supine (head-up) positioning despite the head-up technique being used on a much more critically ill cohort [17]. The supine position is also an independent risk factor associated with widened pulse pressure and decreased diastolic blood pressure [18,19]. Semi-Fowler positioning mitigates posterior atelectasis while potentially being more hemodynamically neutral [13,14]. Given the increased prevalence of obesity among critically ill patients with COVID-19 and their tenuous hemodynamics, we adopted the head-up position for the pre-oxygenation and intubation of these patients [17-20].

Preoxygenation with Bilevel Positive Pressure Ventilation

COVID-19 produces levels of profound hypoxia that were previously rare in patients with normal mentation. Early in the pandemic, many patients underwent early intubation but subsequent observations of increased peri-intubation mortality led to strategies to delay or avoid intubation altogether [21], although most recent data suggest that the timing of intubation has no effect on the morbidity or mortality of critically ill COVID-19 patients [7]. This arguably allows for a more conservative ETI threshold in these patients and for optimization of pre-oxygenation using bi-level positive pressure ventilation [22]. Positive pressure ventilation increases intrathoracic pressure, decreasing preload which may result in hypotension in critically ill patients [23,24]. Despite this known complication, placing profoundly hypoxic patients on positive pressure ventilation prior to induction may maximize alveolar recruitment while simultaneously providing time for anticipation of hypotension. From a logistical standpoint, this further allows team members to address hemodynamics and airway sequentially, rather than simultaneously while attempting emergent ETI.

In comparison to pre-COVID-19 ETI data, critically ill hypoxic patients undergoing ETI demonstrated very high rates of peri-intubation cardiac arrest [6,25]. NEAR registry data evaluating 15,776 patients who experienced cardiac arrest showed that pre-intubation systolic blood pressures of less than 100 mmHg and oxygen saturations less than 90% were both associated with an increased risk of peri-intubation cardiac arrest with adjusted odds ratios of 6.2 and 3.1, respectively [25]. These risk factors appear to be amplified in critically ill patients with COVID-19 [22]. Specifically, pre-intubation hypotension and hypoxemia were also associated with peri-intubation cardiac arrest in 4.2% of patients [26]. The NEAR registry authors note the importance of optimization of pre-intubation oxygenation and hemodynamics, underscoring the importance of a nuanced ETI approach in critically-ill patients, especially in a novel disease such as COVID-19 [25].

Limitations

Our case series evaluated the use of FI optimized by VL, semi-Fowler positioning, and NIPPV preoxygenation to patients with severe COVID-19 requiring mechanical ventilation. It is the result of physicians working within a challenging clinical situation, adapting in real time, and desiring to share lessons learned. Given the retrospective, non-blinded, and non-randomized nature of this study, along with the small cohort, we acknowledge several limitations. First, this study did not have a randomized control group that directly compares outcomes in paired patients with severe COVID-19 requiring mechanical ventilation. Subsequently, our data may be biased in favor of sedation-only intubation. Second, this study was performed at a single tertiary center. While we hope that our cohort, though small, represents the most acute patients with COVID-19, we realize that this study has limited generalizability to other regions of the world. Third, we do not have long-term patient outcomes extending past the study conclusion date. Specifically, while our cohort experienced fewer peri-intubation complications, our study lacks data with respect to in-hospital mortality, total ventilator days, and total hospital days. Fourth, this chart review methodology did not entail double data entry or data collection by multiple data abstractors to facilitate measures of interrater reliability. Fifth, although there were other induction agents used for FI by practitioners prior to RSI becoming the gold standard, we only used midazolam in this study, so it may not be generalizable to other induction agents such as etomidate or propofol. Finally, because current literature does not offer a clear answer as to the optimal patient for facilitated intubation, the decision to pursue FI was at the discretion of the physician, and future studies may benefit from pre-set intubation decision points. 

## Conclusions

In this single-center case series, FI optimized by the use of VL, semi-Fowler positioning, and the use of NIPPV pre-oxygenation, resulted in a 100% first-pass success rate with no cases of peri-intubation cardiac arrest or aspiration. These data are promising and warrant further investigation that perhaps new technologies and techniques have allowed us to overcome the previously established complications associated with FI, and that perhaps FI has a role in the current approach in the emergency airway with a small cohort of patients who are deemed by the intubating physician to be unsuitable for NMBAs.
